# Hollow screw-like drill in plasma using an intense Laguerre–Gaussian laser

**DOI:** 10.1038/srep08274

**Published:** 2015-02-05

**Authors:** Wenpeng Wang, Baifei Shen, Xiaomei Zhang, Lingang Zhang, Yin Shi, Zhizhan Xu

**Affiliations:** 1 State Key Laboratory of High Field Laser Physics, Shanghai Institute of Optics and Fine Mechanics, Chinese Academy of Sciences, P.O. Box 800-211, Shanghai 201800, China

## Abstract

With the development of ultra-intense laser technology, MeV ions can be obtained from laser–foil interactions in the laboratory. These energetic ion beams can be applied in fast ignition for inertial confinement fusion, medical therapy, and proton imaging. However, these ions are mainly accelerated in the laser propagation direction. Ion acceleration in an azimuthal orientation was scarcely studied. In this research, a doughnut Laguerre–Gaussian (LG) laser is used for the first time to examine laser–plasma interaction in the relativistic intensity regime in three-dimensional particle-in-cell simulations. Studies have shown that a novel rotation of the plasma is produced from the hollow screw-like drill of an 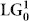 mode laser. The angular momentum of particles in the longitudinal direction produced by the LG laser is enhanced compared with that produced by the usual laser pulses, such as linearly and circularly polarized Gaussian pulses. Moreover, the particles (including electrons and ions) can be trapped and uniformly compressed in the dark central minimum of the doughnut LG pulse. The hollow-structured LG laser has potential applications in the generation of x-rays with orbital angular momentum, plasma accelerators, fast ignition for inertial confinement fusion, and pulsars in the astrophysical environment.

Nowadays, laser intensity can increase up to 10^22^ W/cm^2^ 1,2. Energetic protons have been obtained through different mechanisms, such as target normal sheath acceleration^3^,^4^,^5^,^6^,^7^,^8^,^9^,^10^,^11^,^12^,^13^,^14^,^15^,^16^,^17^, radiation pressure acceleration^18^,^19^,^20^,^21^,^22^,^23^,^24^,^25^,^26^,^27^,^28^,^29^,^30^,^31^,^32^,^33^,^34^,^35^,^36^,^37^, collisionless shock acceleration^38^,^39^,^40^,^41^,^42^, breakout afterburner^43^,^44^, and a combination of different mechanisms^43^,^44^,^45^,^46^. However, these ions are mainly accelerated in the laser propagation direction. Ion acceleration in azimuthal orientation is scarcely mentioned. A circularly polarized (CP) light may carry the angular momentum^47^. The main reason for this phenomenon is that a CP light carries an orbital angular momentum 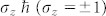 per photon. More than 70 years ago, the mechanical torque created by the transfer of angular momentum of a CP light was first observed in Beth's^47^ experiments. However, the small quantities of the optical angular momentum are difficult to detect in the CP light experiments.

A laser with a Gaussian mode, such as Laguerre–Gaussian (LG) mode, also possesses an orbital angular momentum^48^. A linearly polarized (LP) LG laser with a helical wave-front structure has a central phase singularity^49^. The angular momentum produced by such structure is sometimes referred to an orbital angular momentum, which is different from the spin angular momentum produced by the CP laser pulse^47^. LG laser pulse is circularly symmetric in the cross-section with respect to the optical axis [the direction of light propagation, Figs. 1(b) to 1(e)]. The mode of the LG laser pulse (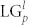) is described by integer indices *l* and *p*, where *l* denotes the number of 2*π* phase cycles around the circumference and (*p* + 1) denotes the number of radial nodes in the mode profile. This study discusses the 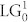 mode, where *l* ≠ 0 indicates the presence of an azimuthal phase term exp(-i*l* *ϕ*) in the laser mode. 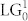 laser carries an orbital angular momentum 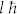 per photon.

The nature of the orbital angular momentum of different LG modes has been investigated in optical trapping experiments. Allen *et al.*^48^ showed that an LG mode has a well-defined orbital angular momentum. They also observed the torque on suspended cylindrical lenses arising from the reversal helicity of an LG mode. He *et al.*^50^ demonstrated that the absorptive particles trapped in the dark central minimum of a doughnut laser pulse are set into rotation. Furthermore, the rotation particles are controlled using both the spin and the orbital angular momentum of light. In such case, the LG light is beneficial because it reduces the ac stark shift and the broadening of transitions at the trap center. Kuga *et al.*^51^ proposed to trap atoms along the beam center using an LG light. They exploited the spatial profile of LG modes with *p* = 0, which has the form of a ring of light. This feature is important in laser cooling and trapping experiments because the repulsive optical dipole force for blue detuned laser light restricts the atoms to the inner dark region of the laser beam, where photon scattering and the associated heating are minimized. Such hollow-structured LG laser can be used to investigate some difficult problems, such as generation of x-rays with orbital angular momentum^52^,^53^,^54^, plasma accelerators^55^,^56^, fast ignition for inertial confinement fusion^57^,^58^,^59^, and pulsars in the astrophysical environment^60^.

## Results

In this letter, the doughnut LG laser is used for the first time in relativistic intensity laser plasma interaction. The LG laser rotates electrons and protons in the azimuthal orientation. Unlike conventional laser pulses, such as LP and CP, enhancement of the proton angular momentum along the longitudinal direction is obtained when an intense LP LG laser pulse irradiates on a thin foil. Three-dimensional (3D) PIC simulations are performed to investigate the LG laser interaction on a foil. In the simulation, the field amplitude *E* (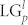) of an LG laser with mode (*l*, *p*) is given by 
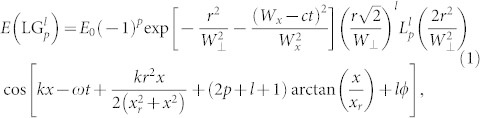
where *E*_0_ is the peak amplitude of the electric field, *r* is the radius, *W*_⊥_ is the radius at which the Gaussian term falls to 1/e of its on-axis value, *W_x_* is the pulse length in the *x* direction, 0 < *t* < 2 *W_x_*/*c*, 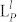 is the generalized Laguerre polynomial, *k* is the wave number, *ω* is the laser frequency, *x* is the distance from the beam waist, *x*_r_ is the Rayleigh range, *x*/*x*_r_ is the Guoy phase of the mode, and ϕ is the azimuthal angle^61^. This study mainly discusses the mode of 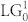, and thus, *p* = 0 and *l* = 1 are used in Eq. (1).

Fig. 2 shows the total angular momentum of the particles (electrons and protons) in 3D PIC simulations. The detailed simulation parameters are shown in the **Methods** section. To describe the rotation effects of 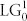 mode on the plasma, the angular momentum of the particles in the *x* direction (the longitudinal direction) *m*_e_ (*yp_z_* − *zp_y_*) + *m*_p_ (*yp_z_* − *zp_y_*) is calculated, where *p_y,_*_*z*_ = *γv_y,_*_*z*_ is the velocity and 
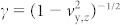
 is the relativistic factor. Fig. 2 further shows the simulation results at different times. The LG laser irradiates on the foil at *t* = 30 *T*, and is totally reflected by the foil at *t* ~ 54 *T*. The angular momentum of the electrons and protons increases up to −1.55 × 10^−17^ kg·m^2^/s until the laser pulse is totally reflected. Thus, the trapped particles rotate by the helicity of the 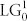 laser.

Fig. 3 shows the angular momentums of particles at 60 *T* for different laser amplitudes *a*_0_. The radius *W*_⊥_ and duration 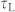 of the laser pulse remain constant for different *a*_0_. The angular momentums increase to −1.32 × 10^−16^ kg·m^2^/s for *a*_0_ = 14. From Fig. 3, a critical condition for the rotation of the particles is observed at *a*_0_ ≈ 5. Detailed simulations have proved this condition. The LG laser is almost reflected for *a*_0_ = 1 at *t* = 50 *T* (Fig. 4). The laser transmits through the foil at *a*_0_ = 5, and more portions of the laser pulse transmit at *a*_0_ = 10. Thus, the critical condition for the proton rotation may be related to the transmission of the foil^18^,^35^,^36^,^62^,^63^,^64^,^65^, and may be expressed by *a*_0_ ≈ *πnd*^35^,^36^,^37^, where the foil density *n* is normalized by *n*_c_ and the foil thickness *d* is normalized by laser wavelength *λ*. The theory estimates that the laser starts to transmit through the foil at *a*_0_ ~ 6.28 for *n* = 2 and *d* = 1 according to *a*_0_ ~ *πnd*, which is larger than the simulation results in Figs. 4(d) to 4(f), where the laser begins to transmit through the foil at *a*_0_ = 5. This phenomenon is attributed to the enhancement of the transmission of the laser pulse caused by the self-focusing of the laser pulse and multi-dimensional instabilities, as demonstrated in our previous simulation study^35^,^36^. Fig. 3 shows that the angular momentums of particles increase when the LG laser transmits through the foil. The main reason is that more laser energy is absorbed by the foil when the laser transmits through the foil. In realistic interaction, the laser transmission may be realized by LG laser pulses with larger intensity and a larger angular momentum of the foil can be generated, similar to the case in Fig. 3.

Fig. 5 shows the distribution of electric fields, electron densities, and ion densities in the (*y*, *z*) plane inside the foil. The corresponding distributions in the (*x*, *y*) plane are shown in Figs. 4(g) to 4(i). The particles (electrons and protons) are rotated in the direction of the helicity of the beam at different position in the foil, just as shown in Fig. 5. The electrons in a ring are drilled out while a compressed point remains at the center at *t* = 50 *T* [Figs. 5(d) to 5(f)]. Such ring structure is related to the helical structure of the 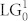 mode laser [Figs. 5(a) to 5(c)]. The trapping and compression of the foil at *x* = 30.5 μm are presented at different time window in Fig. 6, which clearly show the process of trapping and compression with time. Fig. 6(a) shows that the electrons are first dragged along the tangential direction at *t* = 35 *T*. The protons remains almost at rest due to their large mass [Fig. 6(e)]. The protons begin to be accelerated by the charge separation electric field between the electrons and protons as LG laser continues to rotate in the foil [Figs. 6(f)–6(h)]. At *t* = 50 *T*, both the electrons and protons are trapped and compressed into one point by the hollow-structured LG laser. In addition, some ripples are generated at the edge of the ring structure [Figs. 6(d) and 6(h)], which confirms that the 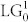 mode laser drills in the plasma like a screw. Such hollow screw-like drill can uniformly trap and compress the plasma at the center [Figs. 5(e) and 5(h)], which may realize the screw-like drilling in the inertial confinement fusion and laser-driven particle accelerations.

## Discussion

The particles are rotated by the LG laser (Fig. 2). The transfer of angular momentum from the laser to the particles is then theoretically estimated in realistic cases. In terms of quantum mechanics, the rotation can be caused by the angular momentum of photons. The LG mode can be seen as the eigenmode of the angular momentum operator of *L*_z_^48^ and carries an orbital angular momentum of 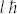 per photon. The angular momentum carried by a photon of a polarized LG mode laser is 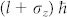, where *σ_z_* is ±1 for the CP laser and 0 for the LP laser. The total angular momentum absorbed from the laser can then be approximately expressed as 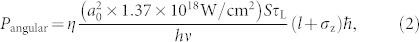
where *η* is the absorbing ratio from the laser pulse during the interaction, 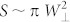 and *W*_⊥_ are the radius at which the Gaussian term falls to 1/*e* of its on-axis value, *h* = 6.63 × 10^−34^ J·s is Plank constant, *ν* = 1/*T* is the frequency of light, *hν* is the energy of single photon, and 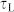 is the pulse duration. *P*_angular_ = 8.22 × 10^−16^ kg·m^2^/s is then obtained for an LP 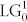 laser (*l* = 1 and *σ_z_* = 0) with *a*_0_ = 10, *W*_⊥_ = 4 μm, and 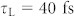. A ring of the target (the radius of the inner and outer ring is 2 and 4 μm, respectively) is assumed to be rotated by the hollow structure of the 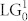 mode laser. Assuming that the angular momentum of the laser is totally transferred to the foil (*η* *= * 1), 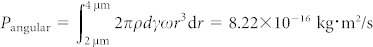 is obtained, where the foil density is 2 × 10^3^ kg/m^3^, foil thickness is *d* = 1 μm, *γ* is the relativistic factor, *ω* is the angular velocity of the foil, and *r* is the radius of the foil. Afterward, *ω* ≈ 1.09 × 10^12^ rad/s is obtained and the velocity of the particle at *r* = 4 μm is approximately 4.36 × 10^6^ m/s. The angular momentum is proportional to the laser amplitude 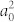 and laser duration 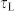, indicating that the angular momentum absorbed from the LG laser can be increased to a certain extent using a high-intensity long pulse based on Eq. (2). Notably, the increase of the angular momentums of particles mainly depends on the laser energy and the absorbing ratio from the laser pulse during the interaction *η*, as shown in Eq. (2). Clearly, a more accurate measurement is taken into account, which states that the total angular momentum is divided by the laser energy. *P*_angular_/*P*_laser_∝*η* is obtained when the laser energy is considered as 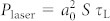. Previous studies have shown that hole boring is deeper for a higher *a*_0_, and the time of hole boring is longer for a larger 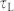
36,37. The particles can absorb more energy from the laser pulse (corresponding to a larger *η*) with the enhancement of the hole boring. Then, a larger *P*_angular_ is obtained according to Eq. (2). It should be noted that the total angular momentum transferred from laser to particles can be calculated from Eq. (2) when the laser pulse does not transmit the foil. In this case, the angular momentum *P*_angular_ is proportional to the laser energy absorbed by the foil (*η* 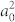 *S* 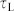). Less angular momentum may be generated when the foil is totally destroyed. In this case, a smaller *η* is obtained and less energy of the LG laser is contributed to total angular momentum of protons.

**Figure 1:**
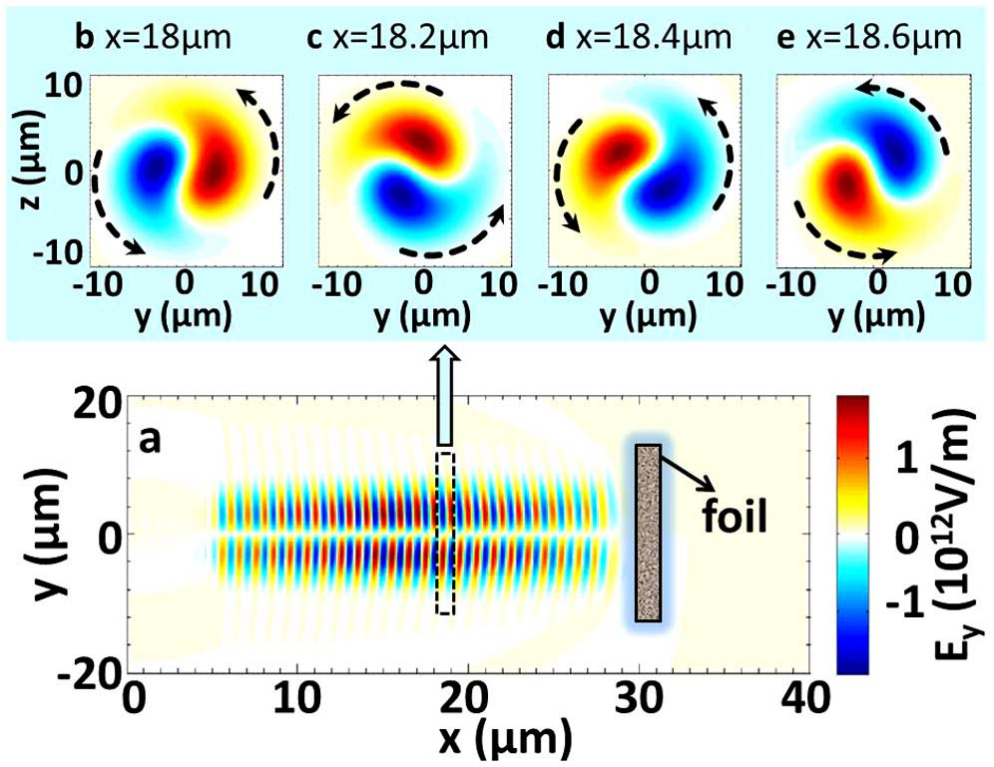
(a) Schematic arrangement of the 3D PIC simulation. The distributions of electric fields in the (*y*, *z*) plane at (b) *x* = 18 μm, (c) *x* = 18.2 μm, (d) *x* = 18.4 μm, and (e) *x* = 18.6 μm.

**Figure 2:**
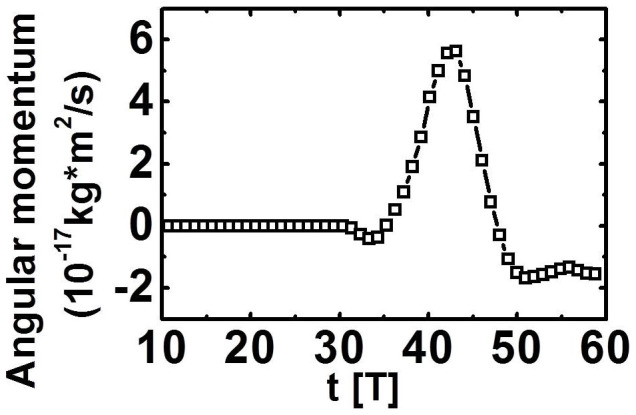
Total angular momentums *m*_e_ (*yp_z_* − *zp_y_*) + *m*_p_ (*yp_z_* − *zp_y_*) in the *x* direction.

**Figure 3:**
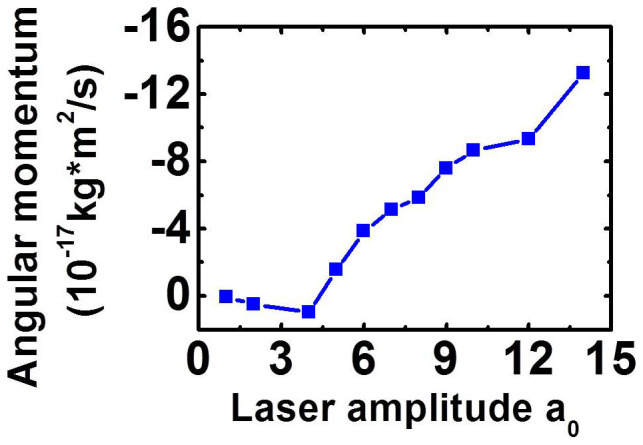
Angular momentums of particles at 60 *T* for different laser amplitudes *a*. The initial foil density is *n*_0_ = 2 *n*_c_ and the foil thickness is *d* = 1 μm.

**Figure 4:**
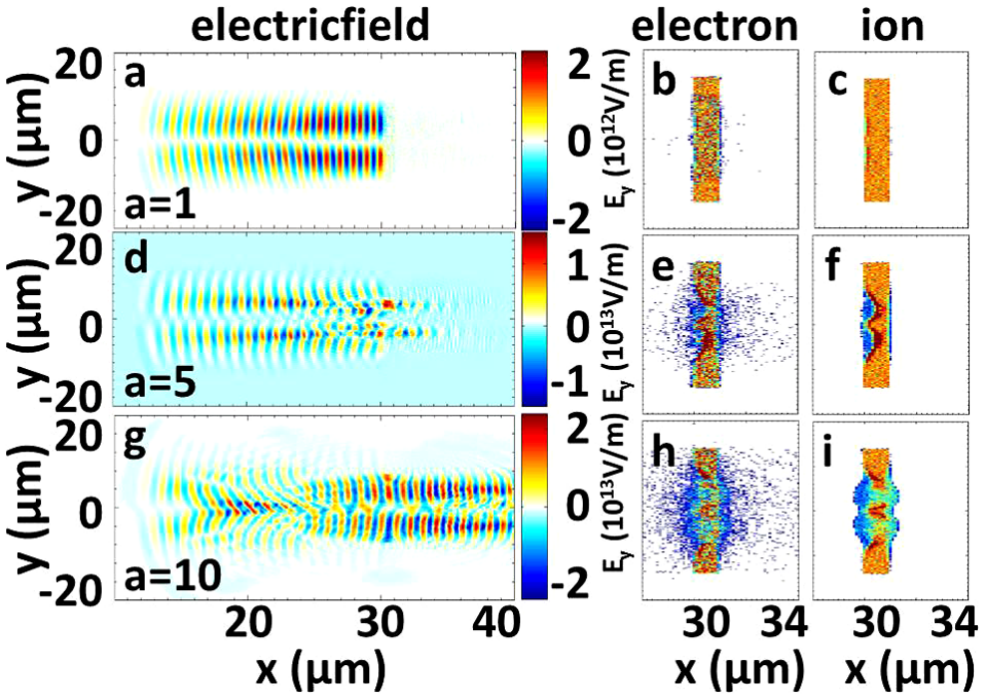
LG laser interactions on the foils for (a–c) *a* = 1, (d–f) *a* = 5, and (g–i) *a* = 10. The distributions of the electric fields (first column), electron density (middle column), and ion density (third column) are shown at *t* = 50 *T*.

**Figure 5:**
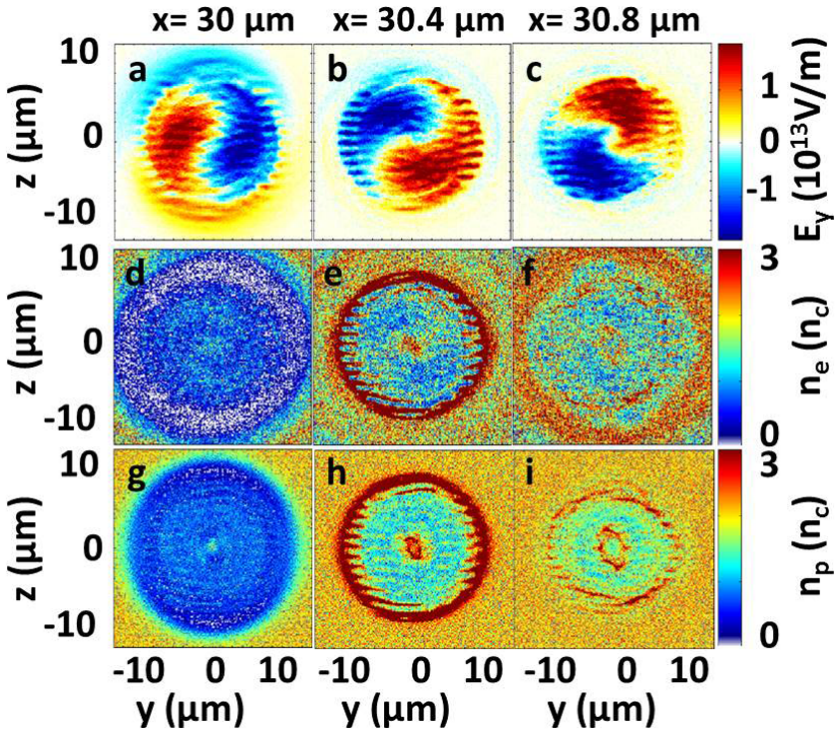
Distributions of (a–c) electric fields, (d–f) electron densities, and (g–i) ion densities in the (*y*, *z*) plane at *x* = 30 μm (first column), *x* = 30.4 μm (second column), and *x* = 30.8 μm (third column) at *t* = 50 *T*. The corresponding distributions in the (x , y) plane are shown in Figs. 4(g) to 4(i).

**Figure 6:**
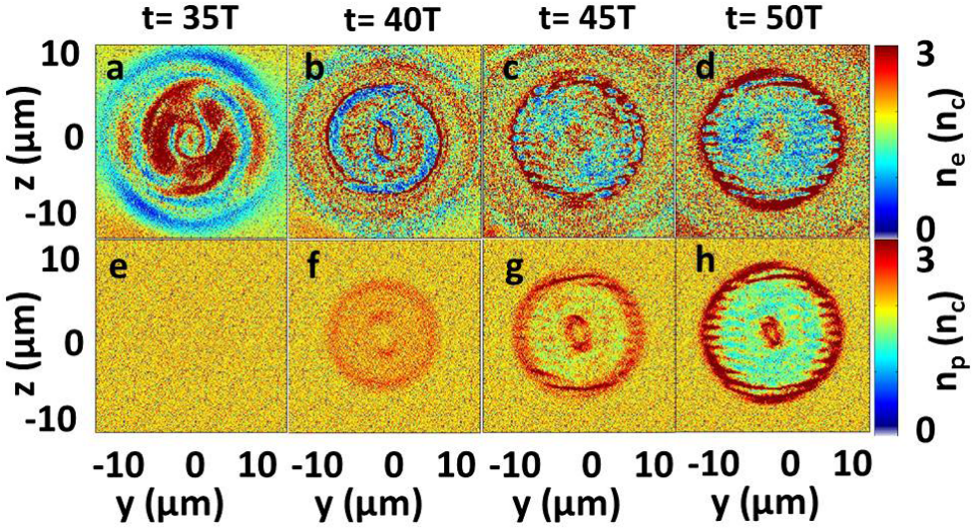
Distributions of (a–d) electron densities and (e–h) ion densities in the (*y*, *z*) plane at *x* = 30.5 μm for different time *t* = 35 *T*, 40 *T*, 45 *T*, and 50 *T*.

**Figure 7:**
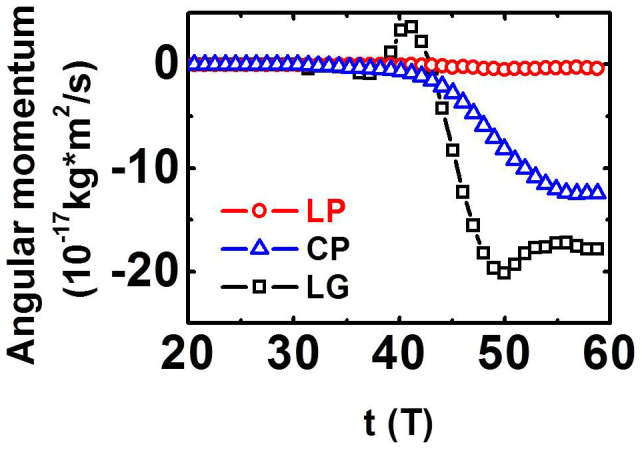
Total angular momentums of protons in the longitudinal direction for LG (squares, *a* = 10), LP (circles, *a* = 7.4), and CP (triangles, *a* = 5.2) laser pulses at *t* = 60 *T*; *d* = 1 μm, and *n*_0_ = 4 *n*_c_.

To show the difference of LG laser on the rotation of the particles, LP and CP laser pulses are also considered. The total angular momentum density per unit power has been defined by Allen *et al.*, where the cases of LP (*σ_z_* = 0) and CP (*σ_z_* = ±1) are considered^48^. Fig. 7 shows that the angular momentums of the particles in the longitudinal direction *m*_e_ (*yp_z_* − *zp_y_*) + *m*_p_ (*yp_z_* − *zp_y_*) for LG, LP, and CP laser pulses. The amplitude of the LP pulse is 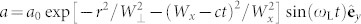 (*a*_0_ = 7.4), and the amplitude of the CP pulse is 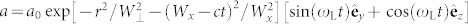 (*a*_0_ = 5.2), where 0 < *t* < 2 *W_x_*/*c*. The amplitude of the LG pulse is expressed by Eq. (1), where *a*_0_ = 10, *W*_⊥_ = 4 μm, and *W_x_* = 12 μm; *d* = 1 μm, and *n*_0_ = 4 *n*_c_ are used in three cases. The values of laser energy in three cases are similar. Compared with LP and CP lasers, the LG laser can generate larger angular momentum of particles (Fig. 7), indicating that the LG laser is beneficial for the rotation of the particles. Compared with case of CP, the doughnut-structured LG pulse (see Fig. 1) has an S-shape potential well just as shown in Fig. 1. More electrons and ions can be trapped in the potential well as the LG laser screw-like drill into the plasma. The LG laser also works as a fan to blow the plasmas forward, which may enhance the absorption of the laser pulse *η* (Fig. 5). Thus the total angular momentums of protons can be raised, just as shown in Fig. 7.

In conclusion, the particles rotation alignment in the tangential direction is realized with the use of an intense LG laser pulse. Compared with LP and CP lasers, the enhancement of the proton angular momentum in the longitudinal direction is obtained when an intense LP LG laser pulse irradiating on a thin foil. The PIC simulations show that the LG pulse can drill into the plasma like a screw. The angular momentum of the particles produced by the LG laser is enhanced compared with that produced by the LP and CP pulses with similar pulse energies. It is also found that electrons and protons are trapped and uniformly compressed in the dark central minimum of the doughnut LG pulse. LG laser has been generated by several techniques. For example, a high-order Hermite–Gaussian (HG) mode can be generated by inserting an intra-cavity cross-wire into a laser cavity. An LG laser can then be obtained using a mode converter on this HG laser^66^. A spiral phase plate^67^ and a computer-generated hologram^49^ may be used to generate the LG modes from a fundamental Gaussian mode (TEM_00_). The intense LG laser exhibits potential applications in field of relativistic intensity, such as laser-driven plasma accelerators. Such LG laser can be applied in the TNSA or RPA experiments to generate a particle beam with angular momentum in the future.

## Methods

The 3D simulations are performed with VORPAL, which is a relativistic, arbitrary, and dimensional hybrid plasma and beam simulation code. It includes the utilities for data analysis and scripts for data visualization. Particle-in-cell (PIC) algorithm is used in VORPAL to describe the kinetic plasma model. A charge-conserving current deposition algorithm is applied in electromagnetic limit to enable the integration of Maxwell's equations without any additional divergence correction. The instantaneous charge distribution is used to calculate Poisson's equation at every time step in the electrostatic limit.

In our simulation, a 40 fs *p*-polarized 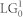 laser pulse is incident from the left side on the foil. The dimensionless peak amplitude of the incident laser pulse is *a*_0_ = *eE*_0_/*m*_e_ω_L_*c* = 5 (the intensity is *I* = 3.4 × 10^19^ W/cm^2^), where ω_L_ is the laser frequency, *λ* = 1 μm is the laser wavelength, *c* is the light speed in a vacuum, and *m*_e_ and *e* are the electron mass and charge at rest. The radius of the laser is *W*_⊥_ = 4 μm and *W_x_* = 12 μm. The laser front reaches the front surface of the foil at *t* = 30 *T* (*T* = *λ*/*c*). The foil thickness is *d* = 1 μm and the front surface of the foil is at *x* = 30 μm. The transverse range of the foil is −14 μm < *y* < 14 μm and −14 μm < *z* <14 μm. The foil is assumed to be fully ionized into protons and electrons before the arrival of the main pulse. The foil density is *n*_0_ = 2 *n*_c_, where 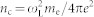 is the critical density. A low-density step-like density profile is used to simplify the model and reduce the 3D PIC simulation time. The size of the simulation box is (60 × 60 × 60) μm, and the cell number is 600 × 600 × 600. Each cell is filled with 10 protons and 10 electrons.

## Author Contributions

W.-P.W. and B.-F.S. contributed to all aspects of this work. B.-F.S., X.-M.Z. and Y.S. provided inspiring ideas to help W.-P.W. write the paper. L.-G.Z. helped in developing the plotting program in the 3D PIC simulations. Z.-Z.X. gave some useful suggestions for this work. All authors discussed the results and commented on the manuscript.

## References

[b1] Chvykov V., Rousseau P., Reed S., Kalinchenko G. & Yanovsky V. Generation of 10^11^ contrast 50 TW laser pulses. Opt. Lett. 31, 1456 (2006). 16642137 10.1364/ol.31.001456

[b2] Yanovsky V. *et al.* Ultrahigh intensity 300 TW laser at 0.1 Hz repetition rate. Optics Express 16, 2109 (2008). 18542290 10.1364/oe.16.002109

[b3] Schwoerer H. *et al.* Laser-plasma acceleration of quasi-monoenergetic protons from microstructured targets. *Nature*. 439, 445 (2006). 10.1038/nature04492 16437110

[b4] Hegelich B. M. *et al.* Laser acceleration of quasi-monoenergetic MeV ion beams. Nature 439, 441 (2006). 16437109 10.1038/nature04400

[b5] Clark E. L. *et al.* Measurements of Energetic Proton Transport through Magnetized Plasma from Intense Laser Interactions with Solids. Phys. Rev. Lett. 84, 670 (2000). 11017343 10.1103/PhysRevLett.84.670

[b6] Maksimchuk A., Gu S., Flippo K. & Umstadter D. Forward Ion Acceleration in Thin Films Driven by a High-Intensity Laser. Phys. Rev. Lett. 84, 4108 (2000). 10990622 10.1103/PhysRevLett.84.4108

[b7] Snavely R. A. *et al.* Intense High-Energy Proton Beams from Petawatt-Laser Irradiation of Solids. Phys. Rev. Lett. 85, 2945 (2000). 11005974 10.1103/PhysRevLett.85.2945

[b8] Wilks S. C. *et al.* Energetic proton generation in ultra-intense laser-solid interactions. Phys. Plasmas 8, 542 (2001).

[b9] Mackinnon A. *et al.* Enhancement of Proton Acceleration by Hot-Electron Recirculation in Thin Foils Irradiated by Ultraintense Laser Pulses. Phys. Rev. Lett. 88, 215006 (2002). 12059483 10.1103/PhysRevLett.88.215006

[b10] Cowan T. E. *et al.* Photonuclear Fission from High Energy Electrons from Ultraintense Laser-Solid Interactions. Phys. Rev. Lett. 84, 903 (2000). 11017401 10.1103/PhysRevLett.84.903

[b11] Kaluza M. *et al.* Influence of the Laser Prepulse on Proton Acceleration in Thin-Foil Experiments. Phys. Rev. Lett. 93, 045003 (2004). 15323768 10.1103/PhysRevLett.93.045003

[b12] Fuchs J. *et al.* Laser-driven proton scaling laws and new paths towards energy increase. Nat. Phys. 2, 48 (2006).

[b13] Robson L. *et al.* Scaling of proton acceleration driven by petawatt-laser–plasma interactions. Nat. Phys. 3, 58 (2007).

[b14] Neely D. *et al.* Enhanced proton beams from ultrathin targets driven by high contrast laser pulses. Appl. Phys. Lett. 89, 021502 (2006).

[b15] Gaillard S. A. *et al.* Increased laser-accelerated proton energies via direct laser-light-pressure acceleration of electrons in microcone targets. Phys. Plasmas 18, 056710–056711 (2011).

[b16] Wang W. P. *et al.* Generation of low-divergence megaelectronvolt ion beams from thin foil irradiated with an ultrahigh-contrast laser. Appl. Phys. Lett. 101, 214103 (2012).

[b17] Wang W. P. *et al.* Effects of nanosecond-scale prepulse on generation of high-energy protons in target normal sheath acceleration. Appl. Phys. Lett. 102, 224101 (2013).

[b18] Shen B. F. & Xu Z. Z. Transparency of an overdense plasma layer. Phys. Rev. E 64, 056406 (2001). 10.1103/PhysRevE.64.056406 11736100

[b19] Esirkepov T., Borghesi M., Bulanov S. V., Mourou G. & Tajima T. Highly Efficient Relativistic-Ion Generation in the Laser-Piston Regime. Phys. Rev. Lett. 92, 175003 (2004). 15169160 10.1103/PhysRevLett.92.175003

[b20] Macchi A., Cattani F., Liseykina T. V. & Cornolti F. Laser Acceleration of Ion Bunches at the Front Surface of Overdense Plasmas. Phys. Rev. Lett. 94, 165003 (2005). 15904236 10.1103/PhysRevLett.94.165003

[b21] Robinson A. P. L., Zepf M., Kar S., Evans R. G. & Bellei C. Radiation pressure acceleration of thin foils with circularly polarized laser pulses. New J. Phys. 10, 013021 (2008).

[b22] Yan X. Q. *et al.* Generating High-Current Monoenergetic Proton Beams by a CircularlyPolarized Laser Pulse in the Phase-StableAcceleration Regime. Phys. Rev. Lett. 100, 135003 (2008). 18517963 10.1103/PhysRevLett.100.135003

[b23] Zhang X. *et al.* Efficient GeV ion generation by ultraintense circularly polarized laser pulse. Phys. Plasmas 14, 123108 (2007).

[b24] Qiao B., Zepf M., Borghesi M. & Geissler M. Stable GeV Ion-Beam Acceleration from Thin Foils by Circularly Polarized Laser Pulses. Phys. Rev. Lett. 102, 145002 (2009). 19392446 10.1103/PhysRevLett.102.145002

[b25] Chen M., Pukhov A., Yu T. P. & Sheng Z. M. Enhanced Collimated GeV Monoenergetic Ion Acceleration from a Shaped Foil Target Irradiated by a Circularly Polarized Laser Pulse. Phys. Rev. Lett. 103, 024801 (2009). 19659213 10.1103/PhysRevLett.103.024801

[b26] Macchi A., Veghini S. & Pegoraro F. "Light Sail" Acceleration Reexamined. Phys. Rev. Lett. 103, 085003 (2009). 19792733 10.1103/PhysRevLett.103.085003

[b27] Yan X. Q., Wu H. C., Sheng Z. M., Chen J. E. & Meyer-ter-Vehn J. Self-Organizing GeV, Nanocoulomb, Collimated Proton Beam from Laser Foil Interaction at 7*10^21^ W/cm^2^. Phys. Rev. Lett. 103, 135001 (2009). 19905516 10.1103/PhysRevLett.103.135001

[b28] Henig A. *et al.* Radiation-Pressure Acceleration of Ion Beams Driven by Circularly Polarized Laser Pulses. Phys. Rev. Lett. 103, 245003 (2009). 20366205 10.1103/PhysRevLett.103.245003

[b29] Bulanov S. V. *et al.* Unlimited Ion Acceleration by Radiation Pressure. Phys. Rev. Lett. 104, 135003 (2010). 20481890 10.1103/PhysRevLett.104.135003

[b30] Yu T.-P., Pukhov A., Shvets G. & Chen M. Stable Laser-Driven Proton Beam Acceleration from a Two-Ion-Species Ultrathin Foil. Phys. Rev. Lett. 105, 065002 (2010). 20867984 10.1103/PhysRevLett.105.065002

[b31] Tripathi V. K., Liu C. S., Shao X., Eliasson B. & Sagdeev R. Z. Laser acceleration of monoenergetic protons in a self-organized double layer from thin foil. Plasma Physics and Controlled Fusion 51, 024014 (2009).

[b32] Robinson A. P. L. Production of high energy protons with hole-boring radiation pressure acceleration. Phys. Plasmas 18, 056701 (2011).

[b33] Liseikina T. V. & Macchi A. Features of ion acceleration by circularly polarized laser pulses. Appl. Phys. Lett. 91, 171502–171503 (2007).

[b34] Zhuo H. B. *et al.* Quasimonoenergetic Proton Bunch Generation by Dual-Peaked Electrostatic-Field Acceleration in Foils Irradiated by an Intense Linearly Polarized Laser. Phys. Rev. Lett. 105, 065003 (2010). 20867985 10.1103/PhysRevLett.105.065003

[b35] Wang W. P. *et al.* Efficient acceleration of monoenergetic proton beam by sharp front laser pulse. Phys. Plasmas 18, 013103 (2011).

[b36] Wang W. P. *et al.* Dynamic study of a compressed electron layer during the hole-boring stage in a sharp-front laser interaction region. Phys. Rev. ST Accel. Beams 15, 081302 (2012).

[b37] Wang W. P. *et al.* Ion motion effects on the generation of short-cycle relativistic laser pulses during radiation pressure acceleration. High Power Laser Sci. Eng. 2, e9 (2014).

[b38] Silva L. O. *et al.* Proton Shock Acceleration in Laser-Plasma Interactions. Phys. Rev. Lett. 92, 015002 (2004). 14753995 10.1103/PhysRevLett.92.015002

[b39] d'Humières E., Lefebvre E., Gremillet L. & Malka V. Proton acceleration mechanisms in high-intensity laser interaction with thin foils. Phys. Plasmas 12, 062704 (2005).

[b40] Chen M. *et al.* Ion acceleration by colliding electrostatic shock waves in laser-solid interaction. Phys. Plasmas 14, 113106 (2007).

[b41] He M.-Q. *et al.* Acceleration dynamics of ions in shocks and solitary waves driven by intense laser pulses. Phys. Rev. E 76, 035402 (2007). 10.1103/PhysRevE.76.035402 17930299

[b42] Fiuza F. *et al.* Laser-Driven Shock Acceleration of Monoenergetic Ion Beams. Phys. Rev. Lett. 109, 215001 (2012). 23215596 10.1103/PhysRevLett.109.215001

[b43] Shen B. F., Zhang X. M., Sheng Z. M., Yu M. Y. & Cary J. High-quality monoenergetic proton generation by sequential radiation pressure and bubble acceleration. Phys. Rev. ST Accel. Beams 12, 121301 (2009).

[b44] Yu L.-L. *et al.* Generation of tens of GeV quasi-monoenergetic proton beams from a moving double layer formed by ultraintense lasers at intensity 10^21^–10^23^ Wcm^−2^. New J. Phys. 12, 045021 (2010).

[b45] Zheng F. L. *et al.* An ultra-short and TeV quasi-monoenergetic ion beam generation by laser wakefield accelerator in the snowplow regime. EPL (Europhysics Letters) 95, 55005 (2011).

[b46] Wang W. P. *et al.* Cascaded target normal sheath acceleration. Phys. Plasmas 20, 113107 (2013).

[b47] Beth R. Mechanical Detection and Measurement of the Angular Momentum of Light. Phys. Rev. 50, 115–125 (1936).

[b48] Allen L., Beijersbergen M., Spreeuw R. & Woerdman J. Orbital angular momentum of light and the transformation of Laguerre-Gaussian laser modes. Phys. Rev. A 45, 8185–8189 (1992). 9906912 10.1103/physreva.45.8185

[b49] Heckenberg N. R., G. McDuff R., Smith C. P., Rubinsztein-Dunlop H. & Wegerner M. J. Laser beams with phase singularities. Opt. Quant. Electron. 24, S951 (1992).

[b50] He H., Friese M., Heckenberg N. & Rubinsztein-Dunlop H. Direct Observation of Transfer of Angular Momentum to Absorptive Particles from a Laser Beam with a Phase Singularity. Phys. Rev. Lett. 75, 826–829 (1995). 10060128 10.1103/PhysRevLett.75.826

[b51] Kuga T., Torii Y., Shiokawa N. & Hirano T. Novel Optical Trap of Atoms with a Doughnut Beam. Phys. Rev. Lett. 78, 4713 (1997).

[b52] Hemsing E. & Marinelli A. Echo-Enabled X-Ray Vortex Generation. Phys. Rev. Lett. 109, 224801 (2012). 23368128 10.1103/PhysRevLett.109.224801

[b53] Hemsing E. *et al.* Coherent optical vortices from relativistic electron beams. Nat. Phys. 9, 549 (2013).

[b54] Bahrdt J. *et al.* First Observation of Photons Carrying Orbital Angular Momentum in Undulator Radiation. Phys. Rev. Lett. 111, 034801 (2013). 23909330 10.1103/PhysRevLett.111.034801

[b55] Mendonça J. T. & Vieira J. Donut wakefields generated by intense laser pulses with orbital angular momentum. Phys. Plasmas 21, 033107 (2014).

[b56] Vieira J. & Mendonça J. T. Nonlinear laser driven donut wakefields for positron and electron acceleration. arXiv.org 1404.3963 (2014).

[b57] Tabak M. *et al.* Ignition and high gain with ultrapowerful lasers. Phys. Plasmas 1, 1626 (1994).

[b58] Naumova N. *et al.* Physical Review Letters 102 025002 Hole Boring in a DT Pellet and Fast-Ion Ignition with Ultraintense Laser Pulses. Phys. Rev. Lett. 102, 025002 (2009). 19257282 10.1103/PhysRevLett.102.025002

[b59] Eliezer S. & Pinhasi S. V. Heat wave fast ignition in inertial confinement energy. High Power Laser Sci. Eng. 1, 44 (2013).

[b60] Martin H. Photon Orbital Angular Momentum in Astrophysics. Astroph ys. J. 597, 1266 (2003).

[b61] Clifford M. A., Arlt J., Courtial J. & Dholakia K. High-order Laguerre–Gaussian laser modes for studies of cold atoms. Opt. Commun. 156, 300 (1998).

[b62] Vshivkov V. A., Naumova N. M., Pegoraro F. & Bulanov S. V. Nonlinear electrodynamics of the interaction of ultra-intense laser pulses with a thin foil. Phys. Plasmas 5, 2727–2741 (1998).

[b63] Lai C. S. Strong Transverse Electromagnetic Waves in Overdense Plasmas. Phys. Rev. Lett. 36, 966 (1976).

[b64] Cattani F., Kim A., Anderson D. & Lisak M. Threshold of induced transparency in the relativistic interaction of an electromagnetic wave with overdense plasmas. Phys. Rev. E 62, 1234 (2000). 10.1103/physreve.62.1234 11088582

[b65] Eremin V. I., Korzhimanov A. V. & Kim A. V. Relativistic self-induced transparency effect during ultraintense laser interaction with overdense plasmas: Why it occurs and its use for ultrashort electron bunch generation. Phys. Plasmas 17, 043102 (2010).

[b66] Beijersbergen M. W., Allen L., Veen H. E. L. O. v. d. & Woerdman J. P. Astigmatic laser mode converters and transfer of orbital angular momentum. Opt. Commun. 96, 123 (1993).

[b67] Turnbull G. A., Robertson D. A., Smith G. M., Allen L. & Padgett M. J. The generation of free-space Laguerre-Gaussian modes at millimetre-wave frequencies by use of a spiral phaseplate. Opt. Commun. 127, 183 (1996).

